# Negligible heat strain in armored vehicle officers wearing personal body armor

**DOI:** 10.1186/1745-6673-6-22

**Published:** 2011-07-31

**Authors:** Ian B Stewart, Andrew P Hunt

**Affiliations:** 1 Institute of Health and Biomedical Innovation, Queensland University of Technology 60 Musk Avenue, Kelvin Grove, QLD 4059, Australia

## Abstract

**Objectives:**

This study evaluated the heat strain experienced by armored vehicle officers (AVOs) wearing personal body armor (PBA) in a sub-tropical climate.

**Methods:**

Twelve male AVOs, aged 35-58 years, undertook an eight hour shift while wearing PBA. Heart rate and core temperature were monitored continuously. Urine specific gravity (USG) was measured before and after, and with any urination during the shift.

**Results:**

Heart rate indicated an intermittent and low-intensity nature of the work. USG revealed six AVOs were dehydrated from pre through post shift, and two others became dehydrated. Core temperature averaged 37.4 ± 0.3°C, with maximum's of 37.7 ± 0.2°C.

**Conclusions:**

Despite increased age, body mass, and poor hydration practices, and Wet-Bulb Globe Temperatures in excess of 30°C; the intermittent nature and low intensity of the work prevented excessive heat strain from developing.

## 1. Background

The human body requires a relatively constant core body temperature to function effectively. In order to maintain a stable temperature, the body must continually lose heat to the surrounding environment at the same rate as heat is produced. Several factors influence the capacity for heat exchange to meet the required rate; these include environmental factors (air temperature, wind speed, relative humidity, and radiant heat), metabolic rate, and clothing. In certain occupations the clothing that an individual is required to wear takes on a protective role. These clothing ensembles are generally very effective at preventing injury when a specific hazard is encountered, however, the added weight of the clothing, and its effect on the transfer of body heat to the environment, can significantly increase the heat strain experienced by the wearer [1].

Cash in transit security guards, also known as armored vehicle officers (AVOs), are increasingly being required to wear ballistic protection or personal body armor (PBA). A number of cash in transit companies have introduced these mandatory policies in response to fatalities in the industry. The function of the PBA is to protect the wearer from physical harm if a hostile situation is encountered. In performing this vital role, the effect the PBA has on thermal balance also needs to be considered.

Since the initial investigations thirty years ago [2-5] there has been a scarcity of research conducted into the effects of PBA on heat strain [6-9]. During this period the evolution of the PBA has been significant as improvements in the ballistic properties of materials has resulted in lighter garments. Military studies have revealed that in climatic conditions of 27°C Wet Bulb Globe Temperature (WBGT) or higher, soldiers wearing body armor in addition to their normal uniform show higher heat strain (core and skin temperatures, higher heart rates, and less sweat evaporation) than those only wearing the normal uniform when marching [3-7,9]. The higher heat strain was attributed to reduced evaporation of sweat as the armor was impermeable and covered approximately 30% of the body surface.

PBA utilized by security agencies i.e. police, customs officials and security companies typically covers less surface area than PBA designed for military. To the authors' knowledge only one study exists of the heat strain encountered by security agencies wearing PBA. This study simulated work tasks and indicated that although core temperature and sweat loses were similar between security personnel wearing PBA and those who were not, heart rate and skin temperature were higher, and sweat evaporation was lower when wearing the PBA [8]. Again it was concluded that the moderately higher heat strain was likely due to reductions in evaporative heat loss.

Taken together, previous research shows that wearing PBA has the potential to increase heat strain. Whether it does or not will depend on the climatic conditions in which work is undertaken, and the physical demand of that work. These factors will of course vary from one workplace to the next, and differ with geographical location and season. The purpose of the present investigation was to evaluate the heat strain experienced by AVOs, during an actual shift, while wearing PBA in a sub-tropical climate.

## 2. Methods

### 2.1 Participants

Twelve male armored vehicle officers (AVOs) aged 41 ± 7.9 years, 1.85 ± 0.05 m in height, 107 ± 21.3 kg in body mass, and an estimated VO_2max _of 36 ± 7.7 ml/kg/min volunteered to participate. The procedures carried out in this study were approved by the University Human Research Ethics Committee. Participants were informed of the procedures and had any questions answered to their satisfaction prior to giving their written and oral consent to participant.

### 2.2 Procedures

The day before conducting the monitoring the participants completed a health screen questionnaire and the NASA activity scale [10]. Participants were issued with an ingestible temperature sensor (CorTemp, HQ inc, Palmetto FL, USA) and instructions were given to swallow the sensor the evening prior to monitoring (~ 9 - 10 pm). This was to allow sufficient time for the sensor to pass from the stomach to the intestines, where the reading of core body temperature is optimal [11-13], prior to the beginning of their shift the next morning. All temperature sensors were calibrated [14], the mean difference between sensor temperature and the standard device was 0.01 ± 0.05°C. The linear regression of each sensor was used to correct raw data.

On the day of monitoring, AVOs arrived at base at least 15 minutes prior to the start of their shift wearing their normal uniform (short sleeve shirt, long pants, steel capped boots, and utility belt). A urine sample was collected prior to measurements of height and body mass. AVOs were equipped with a heart rate monitor (Polar S625x, Polar, Kempele, Finland) and a tri-axial accelerometer (Activity Monitor, Alive technologies, Gold Coast, Australia) on their sternum. The data logger for the core body temperature sensor was fastened to the participant's utility belt. Heart rate and core body temperature were simultaneously recorded at one minute intervals. The tri-axial piezo-electric accelerometer (rated to ± 2.4 g) concurrently logged body accelerations in the sagittal, frontal and transverse planes. Acceleration data were sampled at 75 Hz and converted to earth acceleration units (g) based on a prior calibration. Peaks in the vertical acceleration data were used to detect steps as previously reported [15]. Once all physiological monitoring equipment was set up, AVOs donned their PBA (American Body Armor Xtreme^® ^Series, Safariland, Ontario, Canada) before commencing their shift.

The AVOs shift comprised the delivery and collection of cash to small-medium size businesses, banks, and automatic teller machines. Throughout the shift AVOs recorded the times at which they commenced and finished their work tasks. At all other times they remained within their air-conditioned armored vehicle. Urine samples were collected during the shift and kept in an insulated but unchilled container. AVOs also recorded the volume and type of fluids they consumed throughout the shift.

When AVOs returned to base post shift a urine sample was collected. Heart rate and core body temperature recording equipment were removed, and AVOs reported the extent to which any symptoms of heat illness were experienced by completing the heat illness symptoms index [16]. This index rates eleven symptoms of heat illness on a scale from 0 - 10 (0 - no symptom, 3 - mild symptoms that did not interfere with work, 5 - moderate symptoms, 7 - severe symptoms requiring a break from work, 10 - had to stop work).

Monitoring was conducted between February and March with up to two AVOs being monitored per shift. Outdoor climatic conditions, including air temperature and relative humidity, were recorded every 30 minutes between 6 am and 5 pm by the Australian Bureau of Meteorology at its Brisbane weather station (http://www.bom.gov.au/). A weather meter (Kestrel 4000, Kestrel Weather, Australia) was placed inside the armored vehicle and recorded air temperature and relative humidity every 10 minutes.

### 2.3 Analysis

AVOs characteristics including age, height, body mass, gender, and recreational physical activity were used to estimate the participant's maximal rate of oxygen consumption (VO_2max_) using a published prediction equation [17]. Wet Bulb Globe Temperature (WBGT) was estimated from measures of air temperature and relative humidity according to the Australian Bureau of Metrology [18].

Work intensity was estimated as a percentage of Heart Rate Reserve (HRR) [19]. Heart rate and HRR were summarized for both the time spent inside the armored vehicle, and the time spent outside performing work tasks, except in one AVO for whom heart rate data could not be aligned with time of day.

Urine samples collected before, during, and after the shift were analyzed for urine specific gravity (USG) to assess hydration status. Urine specific gravity was measured by a digital refractometer (PAL-10s, ATAGO, Tokyo, Japan).

Several technical difficulties were encountered and resulted in varying amounts of data loss. Six participants had complete core body temperature data; the remaining six had 21, 54, 61, 63, 84, and 88% missing data due to loss of signal. Exclusion of these participants core body temperature did not alter the average or maximum temperatures, only the minimum temperature was 0.1°C lower; therefore all twelve data sets were included in the analysis. Accelerometer data was collected on seven AVOs. Two AVOs did not indicate their fluid consumption or record climatic conditions inside the armored vehicle. A pre-shift urine sample was collected from all twelve AVOs, however one subject was unable to provide a post shift sample. Three AVOs did not provide a mid-shift sample, seven provided a single mid-shift sample, and two provided two mid-shift samples. In these cases, the average USG was taken as the mid-shift value. Eight AVOs had USG data at all time points, and were used in statistical analysis.

Data are summarized as mean and standard deviation unless otherwise indicated. Statistical analysis included independent samples t-test to assess differences in outdoor climatic conditions to those within the armored vehicle. One-way ANOVA with repeated measures was used to assess for differences in USG between pre, mid, and post-shift time points. Paired samples t-test assessed the differences in heart rate and HRR between times spent in the armored vehicle and performing work tasks.

## 3. Results

Shifts commenced between 7:15 - 8:25 am and were 7.76 ± 0.8 hours in duration. On average, 27.9 ± 3.4 work tasks were performed during this time. Work task duration was 8.6 ± 1.8 min, with minimum and maximum durations of 2.3 ± 1.8 and 19.1 ± 4.3 min respectively. A total of 50.1 ± 6.8% of the total shift duration was spent performing work tasks (outside the armored vehicle). The climatic conditions outdoors and inside the armored vehicle are summarized in table 1. Air temperature, relative humidity, and WBGT were significantly higher outdoors.

**Table 1:** Climatic conditions outdoors and within the armored vehicle.

		Outdoor		Armored vehicle			
		*Mean*	*SD*	*Mean*	*SD*	*t*	*p*
							
Average *							
	*Temperature *(°C)	26.9	1.6	21.7	1.0	6.551	**< 0.001**
	*Humidity (%)*	66.7	6.3	53.1	7.2	3.487	**0.006**
	*WBGT *(°C)	28.4	1.6	21.7	1.3	7.597	**< 0.001**
							
**Average maximum^€^**							
	*Temperature *(°C)	29.5	1.5	24.6	1.0	6.194	**< 0.001**
	*Humidity (%)*	88.6	3.8	64.2	8.2	7.003	**< 0.001**
	*WBGT*(°C)	30.1	1.8	24.6	1.3	5.694	**< 0.001**
							
**Maximum^¥^**							
	*Temperature *(°C)	31.2		26.2			
	*Humidity (%)*	93.0		74.5			
	*WBGT *(°C)	33.1		26.1			

* mean values of the entire shift;^€ ^mean of the highest values obtained during each shift;^¥ ^single highest value obtained.

**Figure 1:**
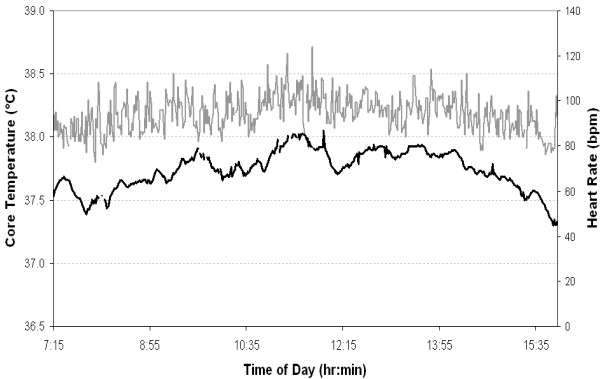
**A representative core body temperature (solid black line) and heart rate (solid grey line) response during a shift**.

Core body temperature and heart rate of a representative AVO is presented in Figure 1. Heart rate and core body temperature throughout the work shift, and separated into time spent inside the armored vehicle and outside, are summarized in table 2. For the six AVOs with complete data, core body temperature increased 0.8 ± 0.2°C during the shift. Average heart rate and HRR were significantly higher when performing work tasks compared to sitting in the armored vehicle (heart rate: t = -6.5, p < 0.001; HRR: t = -6.1, p < 0.001). Average maximum heart rate and HRR were similar between work task and times inside the vehicle (heart rate: t = -2.1, p = 0.057; HRR: t = -2.0, p = 0.070). AVOs accumulated 4528 ± 716 steps over the course of the shift. The number of steps was also separated into clusters (a series of steps separated by periods of no movement). Steps per cluster averaged 16 ± 3.4 with a range from 2 to 192 steps. Cluster duration was 10.5 ± 1.5 s with a range of 2 to 108 s.

**Table 2:** AVOs core temperature and heart rate

	*Mean*	*SD*	Maximum Mean*	*SD*
**Core temperature (°C)**	37.4	0.3	37.7	0.2
				
**Whole shift HR^€ ^(bpm)**	89.6	10.8	129.5	17.0
				
**Task^£ ^HR^€ ^(bpm)**	92.4	10.2	128.7	18.6
				
**Vehicle^§ ^HR^€ ^(bpm)**	85.8	12.1	123.2	18.8
				
**Whole shift HRR^¥ ^(%)**	22.1	9.1	58.0	20.3
				
**Task^£ ^HRR^¥ ^(%)**	24.9	8.8	57.4	21.9
				
**Vehicle^§ ^HRR^¥ ^(%)**	19.4	10.7	52.3	22.9
				

* mean of the absolute maximum value obtained by each AVO; **^€ ^**heart rate; **^¥^**heart rate reserve; **^£^**AVO outside of vehicle undertaking work; **^§ ^**AVO inside vehicle

There was no significant change in urine specific gravity across the shift (1.021 ± 0.005, 1.015 ± 0.007, and 1.021 ± 0.005; pre, mid, and post-shift respectively, F = 2.906, p = 0.088). AVOs reported 2.1 ± 0.8 L of fluid consumption over the shift. Total fluid consumed by each AVO was from a variety of fluid types. Ninety-two percent consumed water, 42% consumed a carbohydrate-electrolyte beverage, 25% soft drink, 8% juice, and 8% protein shake.

Symptoms of heat illness experienced by AVOs during the shift are summarized in table 3. Three AVOs reported no symptoms of heat illness. The remaining nine AVOs reported between one and eight symptoms (mean ± SD = 3.3 ± 2.2).

**Table 3:** Heat illness symptoms experienced

	AVOs Reporting Symptom (%)	Severity*	
		*Mean*	*SD*
**Feeling tired**	83	3.5	1.8
**Cramps**	8	2.0	0.0
**Nausea**	8	2.0	0.0
**Dizziness**	17	3.0	0.0
**Thirst**	58	5.1	3.1
**Vomiting**	0		
**Confusion**	0		
**Muscle weakness**	33	3.3	2.1
**Heat sensations**	42	2.2	1.3
**Chills**	0		
**Feeling light headed**	25	1.7	1.2

* 0 - No symptoms, 3 - mild symptoms that did not interfere with work,

5 - moderate symptoms, 7 - severe symptoms requiring a break from work,

10 - had to stop work

## 4. Discussion

The prevailing weather conditions have a significant impact on the heat stress individuals are exposed to during work. High air temperatures and humidity, and low wind speeds, will slow the rate of heat loss from the body. Work in such conditions can lead to excessive fluid loss through sweating and elevations in core body temperature. In the present investigation the WBGT outside the armored vehicle averaged 28.4°C throughout the day, with peaks over 30°C. Previous research has shown that heat strain is increased at WBGT > 27°C when wearing body armor [3-5,7,9]. Therefore, the outdoor climatic conditions the AVOs were exposed to during the present investigation provided a significant level of heat stress.

The International Organisation for Standardisation (ISO) recommends that work should cease at WBGT ≥ 30°C for acclimatized workers performing continuous low to moderate intensity work [20]. AVOs were however not continuously exposed to this high level of heat stress. Instead, it was an intermittent exposure, whereby AVOs regularly moved between an air-conditioned armored vehicle and the outdoors. The average time spent outdoors before returning to the armored vehicle was 8.6 min, and overall, half of the shift duration was spent outdoors. WBGT was significantly lower inside the armored vehicle (Table 1). Therefore, the time spent inside the armored vehicle reduces heat stress imposed by the climatic conditions over the course of the shift.

The intensity of work tasks is also an important contributor to heat stress in the work environment. Heart rate in the present investigation suggests that the work tasks of AVOs are of a low intensity (Table 2). While inside the vehicle AVOs would have been either standing, (re)positioning cash canisters/satchels, or seated (driving or resting). Performing work tasks involved walking (of durations between 10-120 seconds) carrying various loads and standing. The significantly higher heart rates and HRR when performing work tasks outdoors can primarily be attributed to the higher physical demand of the tasks, but may also be influenced by the heightened anticipation of potential threats and exposure to the heat [21].

Figure 1 highlights the fluctuating nature of core temperature throughout the day. These fluctuations result from job tasks being of varied duration, time between jobs being irregular and the intermittent exposure to the outside environment; as well as the diurnal variation in core temperature [22]. These results (Figure 1 and Table 2) support the assertion that the intermittent nature of exposure, interspersed with breaks in a cooler climate, enables heat strain to stay within reasonable limits. The Australian Institute of Occupational Hygienists (AIOH) [23], ISO 9886 (2004) [24] and ISO 12894 (2001) [25] recommend that core body temperature should not exceed 38.5°C for medically selected and acclimatized personnel. No individual recorded a core temperature in excess of 38.5°C during the present study. The single highest temperature attained was 38.27°C. As such the addition of PBA to the work uniform of AVOs does not appear to increase heat strain to excessive levels.

Hydration status in the current investigation was assessed by collecting urine samples from the participants before, during, and after their working shift. Samples were assessed for specific gravity, with a value of 1.020 or higher being associated with the detrimental effects of dehydration [26,27]. Applying this limit to the present study, six of the twelve participants were dehydrated prior to commencing work. The specific gravity measurements suggest that hydration status improved during the shift; however this does not indicate an overall improvement in all of the participants, as the hydrated individuals provided significantly more urine samples than those who were dehydrated. Post-shift measurements on all participants indicated that six subjects were still classified as dehydrated. In support of these levels of dehydration thirst was also the highest rated symptom reported (Table 3). These findings indicate that despite all vehicles being supplied with cool water and electrolytes that individuals were not drinking sufficient amounts of fluid, even when thirsty. Poor hydration practices should be addressed due to the potential for dehydration to exacerbate heat strain [28,29].

When a group of individuals is exposed to the same hot environment and workload, a variety of physiological responses will be observed. One of the main factors that determine an individual's tolerance to work in a hot environment is their aerobic fitness [30,31]. The use of an activity questionnaire, indicated that only three subjects had an aerobic fitness rating less than "fair". However the almost universal experience of tiredness (Table 3) following the low intensity work performed (Table 2), in combination with five subjects having a body mass in excess of 110 kg, indicates that the self-report fitness levels may have been over-estimated [32].

Higher core body temperatures are often seen in individuals of low fitness and high body mass during heat stress. This is due to a reduced capacity for heat loss through sweating and skin blood flow mechanisms [33,34] and the heat storage qualities of fat tissue [35]. Low fitness and or a high body mass are also commonly found to be risk factors for heat illness [36-38]. With this knowledge in mind, it appears that the AVOs studied provided a representative spread of fitness and body mass levels; including those who would be more susceptible to the adverse effects of heat stress. In spite of this, core temperature remained within safe limits and few symptoms of heat illness were reported (Table 3) and these did not interfere with work. This suggests that the exposure time and WBGT experienced by these workers, plus their level of activity, was insufficient to cause excessive heat strain.

### 4.1 Conclusion

The twelve AVOs whom undertook the physiological monitoring ranged in age from 35-58 years, five had a body mass greater than 110 kg, and three had poor or very poor aerobic fitness. These attributes of age, body mass and fitness level substantially increase the potential risk of heat strain. In conjunction, six AVOs were dehydrated from pre through post shift measurements, and two others became dehydrated across the day, which further exacerbates the potential risk. Despite these personal risk factors and practices, and high WBGT outdoors, none of the AVOs core temperature readings exceeded the 38.5°C level identified as when an individual's exposure to heat stress should be discontinued.

## 5. Competing interests

IBS has received research funding and acted as an advisor to Chubb Security Services, Australia.

## 6. Authors' contributions

IBS designed the study, assisted in data collection, and contributed to the final manuscript. APH undertook data collection and drafted the manuscript. Both authors read and approved the final manuscript.
